# Supplementary material for: Exact firing time statistics of neurons driven by discrete inhibitory noise
Source: Sci Rep. 2017 May 8;7:1577. doi: 10.1038/s41598-017-01658-8 (PMC5431561; doi:10.1038/s41598-017-01658-8)
Supplement: Supplementary file 1 — Supplementary Information [file 41598_2017_1658_MOESM1_ESM.pdf]

# Supplementary Information: Exact firing time statistics of neurons driven by discrete inhibitory noise

Simona Olmi
*Weierstrass Institute for Applied Analysis and Stochastics, Mohrenstraße 39, 10117 Berlin, Germany*
*Aix Marseille Univ, INSERM, INS, Inst Neurosci Syst, Marseille, France and*
*CNR - Consiglio Nazionale delle Ricerche - Istituto dei Sistemi Complessi, 50019 Sesto Fiorentino, Italy*

David Angulo Garcia
*Aix Marseille Univ, INSERM, INS, Inst Neurosci Syst, Marseille, France*
*Aix Marseille Univ, INSERM and INMED, Marseille, France and*
*Aix Marseille Univ, Université de Toulon, CNRS, CPT, UMR 7332, 13288 Marseille, France*

Alberto Imparato
*Aarhus University,Department of Physics and Astronomy, 8000 Aarhus, Denmark*

Alessandro Torcini
*Aix Marseille Univ, INSERM, INS, Inst Neurosci Syst, Marseille, France*
*CNR - Consiglio Nazionale delle Ricerche - Istituto dei Sistemi Complessi, 50019 Sesto Fiorentino, Italy*
*Aix Marseille Univ, INSERM and INMED, Marseille, France*
*CNR - Consiglio Nazionale delle Ricerche - Istituto dei Sistemi Complessi, 50019 Sesto Fiorentino, Italy*
*Aix Marseille Univ, Université de Toulon, CNRS, CPT, UMR 7332, 13288 Marseille, France and*
*Laboratoire de Physique Théorique et Modélisation, CNRS UMR 8089,*
*Université de Cergy-Pontoise, F-95300 Cergy-Pontoise Cedex, France*

## EXPONENTIAL INTEGRATE AND FIRE

The Exponential Integrate-and-Fire (EIF) model is a simple non-linear integrate and fire neuronal model introduced by Fourcaud-Trocmé et al. [1] able to reproduce quite accurately the dynamics of cortical neurons [2]. The model can be written as follows

$$\tau \frac{dv}{dt} = \mu_0 - v + \Delta_T \exp\left(\frac{v - v_{th}}{\Delta_T}\right) + I(t) \ . \tag{1}$$

where $\mu_0$ represents an external DC current and $I(t)$ the synaptic drive. In this model, the spike generation occurs in a finite time controlled by the parameter $\Delta_T$. In particular, once the membrane potential has reached the threshold value $v_{th}$ this will rapidly grow towards infinity in a finite time interval, the parameter $\Delta_T$ establishes how fast the infinite limit is reached. In the limit $\Delta_T \to 0$, the spike generation is instantaneous and the LIF model is recovered. As in the usual LIF model, once the neuron has fired its membrane potential is resetted to the value $v_{re} = 5$ mV, we also set $\tau = 20$ ms and $v_{th} = 10$ mV, as in the LIF model studied in the article.

As a first analysis, we will examine the response of the EIF neuron subject to small Gaussian noise of zero average and intensity $\sigma$, this can be obtained by solving the associated continuity equation for the probability $P(v,t)$ of finding the membrane voltage between $v$ and $v + dv$ at time $t$, which reads as [1]:

$$\frac{\partial P}{\partial t} + \frac{\partial J}{\partial v} = r(t)[\delta(v - v_{re}) - \delta(v - v_{th})] - \delta(t)\delta(v - v_{re}) \tag{2}$$

$$J = \left(\frac{\mu_0 - v + \Delta_T \mathrm{e}^{v - v_{th}/\Delta_T}}{\tau}\right) P - \frac{\sigma^2}{2\tau}\frac{\partial P}{\partial v} \tag{3}$$

where $J = J(v,t)$ is the associated flux. . In this case, since the effective threshold is located at infinity, the steady firing rate can be evaluated as

$$r_0 = \lim_{v \to \infty} J(v) \ ;$$

where $J(v)$ is the stationary solution of the continuity equation for the flux.

In particular, we made use of the threshold-integration method [3] to calculate the firing rate $r_0$ of the EIF neuron sunbject to inhibitory inputs and compare it with the diffusion approximation (DA) for the LIF and the corresponding shot noise solution for $\delta$-distributed IPSP amplitudes with $|a_i| = 0.1$ mV. The results are shown in Fig. 1 (a), where it is clearly shown that for $\Delta_T \to 0$ the EIF results converge to the LIF solution, both for the DA and the the shot noise results found for small IPSP. Furthermore direct simulations of the EIF and the corresponding shot noise solution of the LIF for large IPSP amplitudes, namely $|a_i| = 1$ mV, show that also in this case the LIF limit is recovered for $\Delta_T \to 0$. However it is clear that also in the case of the EIF, the diffusion limit is unable to capture the onset of the activity and the firing rate at small intensities.

[1] Fourcaud-Trocmé N., Hansel D., van Vresswijk C., and Brunel N., How Spike Generation Mechanisms Determine the Neuronal Response to Fluctuating Inputs, *J. Neurosci.* **23** 11628-11640 (2003).

[2] Badel L., Lefort S., Brette R., Petersen C.C.H., Gerstner W.,and Richardson M.J.E., Dynamic I-V curves are reliable predictors of naturalistic pyramidal-neuron voltage traces, *J. Neurophysiol.* **99** 656-666 (2008).

[3] Richardson M.J.E., Firing-rate response of linear and nonlinear integrate-and-fire neurons to modulated current-based and conductance-based synaptic drive, *Phys. Rev. E 76* 021919 (2007).

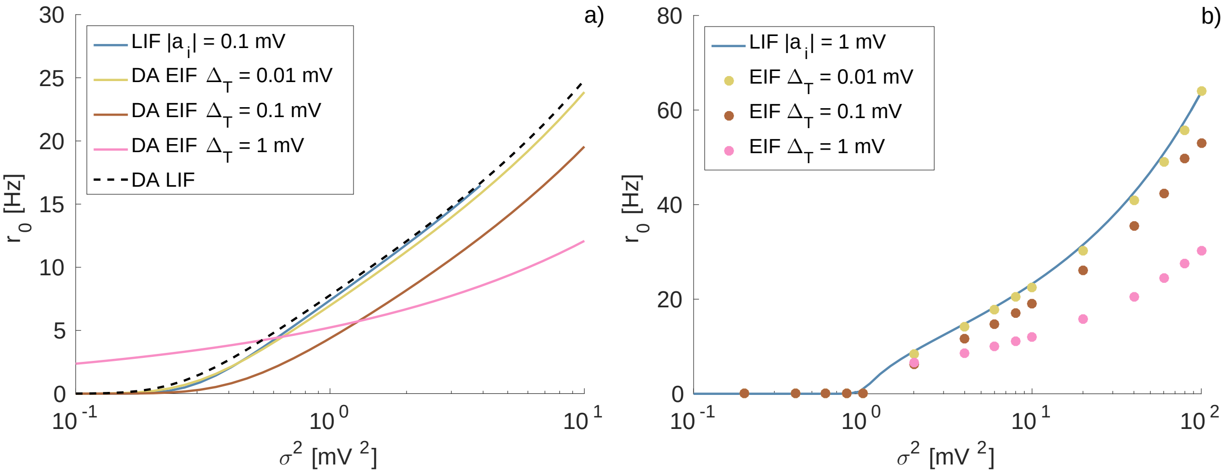


FIG. 1. **Comparison of the firing rate between LIF and EIF** a) Firing rate in the Diffusion limit for the EIF at different $\Delta_T$ and the comparison with the DA and $\delta$-distributed amplitudes of IPSP in the LIF model with small $|a_i| = 0.1$ mV. Results for the EIF are obtained by solving the stationary state of Eqs. (2) and (3) via the threshold integration method as reported in [3]. For the LIF, the DA and the $\delta$-distributed solutions are taken respectively from Eqs. (17) and (51) in the main text. b) Numerical simulations of the EIF in the shot noise case with $\delta$-distributed IPSP amplitudes of average $|a_i| = 1$mV, for the same values of $\Delta_T$ as in panel a), and the corresponding LIF case with the same IPSP distribution. In this panel, the results of the EIF are calculated numerically by integrating Eq. (1) with an Euler scheme with time step $h = 1 \times 10^{-3}$. When the neuron reaches a large value $v_\infty = 80$ mV, the remaining time to reach infinity is calculated as $t_\infty = \tau \exp((v_{th} - v_\infty)/\Delta_T)$ [1]. In all the cases we have chosen $\mu_T = 9$mV, $v_{re} = 5$mV, $v_{th} = 10$mV and $\tau = 20$ms.
